# Protocol to quantify polyphosphate in human cell lines using a tagged PPBD peptide

**DOI:** 10.1016/j.xpro.2022.101363

**Published:** 2022-05-01

**Authors:** Javier Jiménez, Blanca Lázaro, Ana Sarrias, Francisco J. Tadeo, Marta Pérez-Montero, Josep Clotet, Samuel Bru

**Affiliations:** 1 Basic Sciences Department, Universitat International de Catalunya, 08195 Barcelona, Spain

**Keywords:** Cell Biology, Microscopy, Antibody, Protein Biochemistry, Protein expression and purification

## Abstract

Polyphosphate (polyP) is an evolutionarily conserved polymer of phosphates that is difficult to study in human cells because of its low concentration and high lability. First, we described how to express and purify Xpress-tagged PPBD (Ppx1 PolyP Binding Domain). We describe the detection and quantification of nuclear polyP in HEK293T cells using Xpress-PPBD, Xpress antibody, and Alexa-conjugated secondary antibodies. We have also used this protocol in SH-SY5Y HeLa and HEK293 cells.

For complete details on the use and execution of this protocol, please refer to Samper-Martín et al. (2021).

## Highlights

• *E. coli* PPBD peptide to localize polyP in human cells cultures
• PPBD purification and storage is a critical step for polyP staining
• Detection of nuclear polyP fraction that can be degraded by the endopolyPase Nudt3
• Quantification of relative polyP amounts using a free image analysis software

## Graphical abstract

**Figure:**
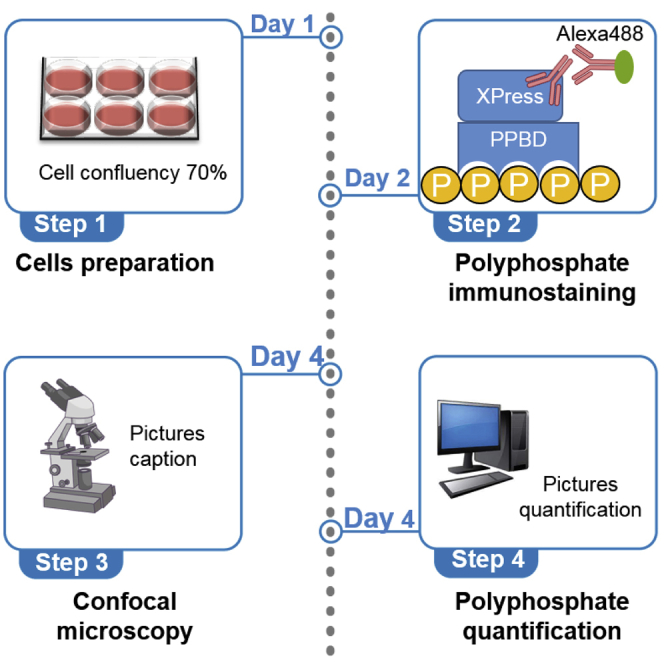

## Before you begin

This protocol describes the steps for obtaining the PPBD peptide (able to bind polyP (Saito et al., 2005)), immunodetecting and quantifying nuclear polyP in HEK293T cells. However, we have also successfully used it in the SH-SY5Y (a brain cell line), HeLa, A549 (lung carcinoma cell line) and HEK293 cell lines. Here we describe details for growing and preparing the cells for polyP detection. We present critical aspects for fixing and permeabilizing the cells. Finally, we describe a method for quantifying the polyP content. Before beginning, it is important to have the recombinant tagged PPBD peptide purified and ready to use (see below).

### Xpress-tagged PPDB peptide cloning, expression, and purification

**Timing: 4 days not including the cloning**

1. PCR amplification of *E.* coli Ppx1 PPBD domain (from residue 306–534) using the oligonucleotides in the key resources table.
2. Cloning PPBD in the pRSET-A expression vector (which includes the 6×His tag for purification and XPress tag). pRSET-A was *Bam*HI-digested and PPBD fragment was cloned using In-Fusion HD Cloning kit. The construct was checked by restriction mapping and sequencing.
3. 6×His-Xpress-PPBD expression and purification:
  a. Transform into BL21 (DE3) pLysS *E.* coli competent cells.
  b. Grow a transformant colony in 50 mL of LB supplemented with ampicillin (50 μg/mL) and chloramphenicol (25 mg/mL) for 16 h at 37°C with agitation.
  c. Add the 50 mL grown culture into 500 mL of fresh LB medium containing the antibiotics as above. Incubate for 2 h at 37°C with agitation.
  d. Add 0.5 mL of 1 M IPTG and incubate for 6 h at 25°C in agitation.
  e. Centrifuge the cells at 3300 rcf for 15 min at 4°C. Remove the supernatant.

**Pause point:** pellet can be stored at −80°C for several weeks.

4. Purification of 6×His-Xpress-PPBD:
  a. Resuspend the cells in the pellet by adding 40 mL of cold lysis buffer (25 mM NaH_2_PO_4_, 150 mM NaCl, 1% (v/v) Triton X-100, 2 mM β-mercaptoethanol, 10 mM imidazole, lysozyme (0.4 mg/mL), 50 Units of benzonase and the protease inhibitors: 1 mM PMSF (phenylmethylsulfonyl fluoride), leupeptin (1 μg/mL), pepstatin (1 μg/mL), and 1 mM benzamidine.
    **CRITICAL:** Lysozyme and benzonase must be added fresh.
  b. Incubate for 15 min at 4°C with gentle agitation. Note that the solution gets less dense and close to transparent.
  c. Sonicate once for 30sec at 30 kHz (Dr. Hielscher, UP 50H).
  d. Centrifuge at 3300 rcf for 10 min at 4°C.
  e. Keep the supernatant and centrifuge at 3300 rcf for 10 min at 4°C. Note that after this second centrifugation the pellet should be very small.
  f. Pipette out the supernatant in a separate tube and dilute it to a final concentration of 2 mg/mL of total protein according to a Bradford quantification.
  g. Purify 6×His-Xpress-PPBD peptide by using a HisTrap HP 5 mL affinity column in an ÄKTA^TM^ start protein purification system. Conditions can be found in the materials and equipment section.
  h. Collect 5 mL from the elution step containing the highest amount of imidazole and inject (1 mL per injection) into an ÄKTA HITrap^TM^ 5 mL desalting column. Desalting buffer was 50 mM Tris-HCl pH 9.
  i. Collect 1 mL aliquot from the fractions 2 and 3 in tubes containing 100 μL glycerol. Repeat in all (five) desalting injections.
    **CRITICAL:** Proceed rapidly to avoid PPBD precipitation.
  j. Concentrate the pooled ÄKTA eluted fractions using Amicon 10 KDa columns until obtaining a volume of 1 mL (around ten folds).
  k. Add glycerol until a final concentration of 50% (v/v) and store at −20°C for several months.
    **CRITICAL:** Different PPBD storage conditions were tested and we chose the one described above.
    ***Note:*** PPBD concentration and purity can be checked by PAGE and coomassie staining. 6×His-XPress-PPBD has a Mw 25kDa.

### Thawing the cells

**Timing: 3 days**

Cells are stored in liquid nitrogen tanks (culture medium containing DMSO 10% (v/v)).

5. Thaw the cells:
  a. Take the vial from the N_2_ tank and thaw in a 37°C water bath. Add to a tube containing 5 mL of pre-warm DMEM supplemented with 10% FBS, 1% glutamax and 1% penicillin/streptomycin (complete DMEM).
  b. Centrifuge at 300 rcf for 3 min. Take the pellet and suspend it in 1 mL of complete DMEM medium.
  c. Add 24 mL of fresh complete DMEM medium into a T175 flask.
  d. Inoculate 1 mL of the recent thaw cells and incubate at 37°C in a cell culture incubator containing CO_2_ 5%.

**CRITICAL:** Cell viability decreases importantly after the thaw when DMSO is present. Proceed rapidly after the thaw.

6. Grow the cells:
  a. After 3 days, and 80%–90% confluence, wash once with 5–10 mL of pre-warmed PBS.
  b. Add 1.5 mL of commercial trypsin solution for 3 min at 37°C in the cell culture incubator.
  c. Harvest the cells by adding 9 mL of fresh culture medium. Put them into a 15 mL tube.
  d. Centrifuge at 300 rcf for 3 min.
  e. Suspend the cells in 5 mL of fresh medium. Take 10% of the cells and seed them in a T75 flask containing 14.5 mL of fresh medium. Incubate at 37°C and 5% CO_2_ in a cell culture incubator.

## Key resources table

**Table:** 

REAGENT or RESOURCE	SOURCE	IDENTIFIER
**Antibodies**		

Mouse monoclonal anti-Xpress	Life Technologies	Cat#46-0528; RRID: AB_2556552
Alexa Fluor 488 conjugated goat anti mouse	Invitrogen	Cat#A11001; RRID: AB_2534069
Alexa Fluor 633 conjugated goat anti mouse	Invitrogen	Cat # A-21052; RRID: AB_2535719

**Bacterial and virus strains**		

*E. coli* DL21 DE3 pLysS	Bio-Rad	Cat#1563003

**Chemicals, peptides, and recombinant proteins**		

PBS 10×	Sigma-Aldrich	Cat# MFCD00131855
Goat serum	Sigma-Aldrich	Cat#G9023
DMEM	Sigma-Aldrich	Cat#D5671
FBS-12A	Quimigen	Cat# FBS-12A
Glutamax	Biowest	Cat#x0551
Trypsin solution	Thermo Fisher Scientific	Cat#25200072
Penicillin-Streptomycin	Sigma-Aldrich	Cat#P0781
L-Lysine solution	Sigma-Aldrich	Cat#P4707
Hoechst 33342	Sigma-Aldrich	Cat#14530
Immersion oil	Leica Microsystems	Cat#195371-10-9
Fluoromount-G	SouthernBiotech	Cat#0100-01
Benzonase	Merck	Cat#103773
Protease inhibitors	Thermo Fisher Scientific	Cat#A32955

**Critical commercial assays**		

In-Fusion HD Cloning kit	Takara Bio	Cat#638920

Cell lines		
HEK293T	ATCC authenticated	N/A

**Oligonucleotides**		

5′GATAAGGATCGATGGGGATCCA TGGAAGGACGTTTCCGTC	Integrated DNA Technologies	Forward
5′GCAGATCTCGAGCTCGGATC CTTAAGCGGCGATTTC	Integrated DNA Technologies	Reverse

**Software and algorithms**		

Fiji ImageJ 1.53c	https://imagej.nih.gov/ij/	Schindelin et al. (2012)

**Other**		

HisTrap HP affinity column	Cytiva	Cat#17524802
ÄKTA HITrapTM 5 mL desalting column	Cytiva	Cat#17140801
pRSET-A	Thermo Fisher Scientific	Cat#V35120
Leica SP8 confocal microscope	Leica Microsystems	N/A
Round 12 mm coverslips	Thermo Scientific	Cat#15820692

## Materials and equipment

**Table:** ÄKTA buffers and conditions

Reagent	Final concentration	Amount
ÄKTA buffer	(25 mM NaH_2_PO_4_, 150 mM NaCl, 2 mM β-Mercaptoethanol	1 mL
Wash buffer	ÄKTA buffer supplemented with 20 mM imidazole	3 times the column volume
Elution buffer (3 buffers differing in the progressive increase in the imidazole concentration)	ÄKTA buffer supplemented with 50, 200, and 500 mM imidazole	Once with each. 2 column volumes each
Column Flow		1 mL/min

**Figure 1:**
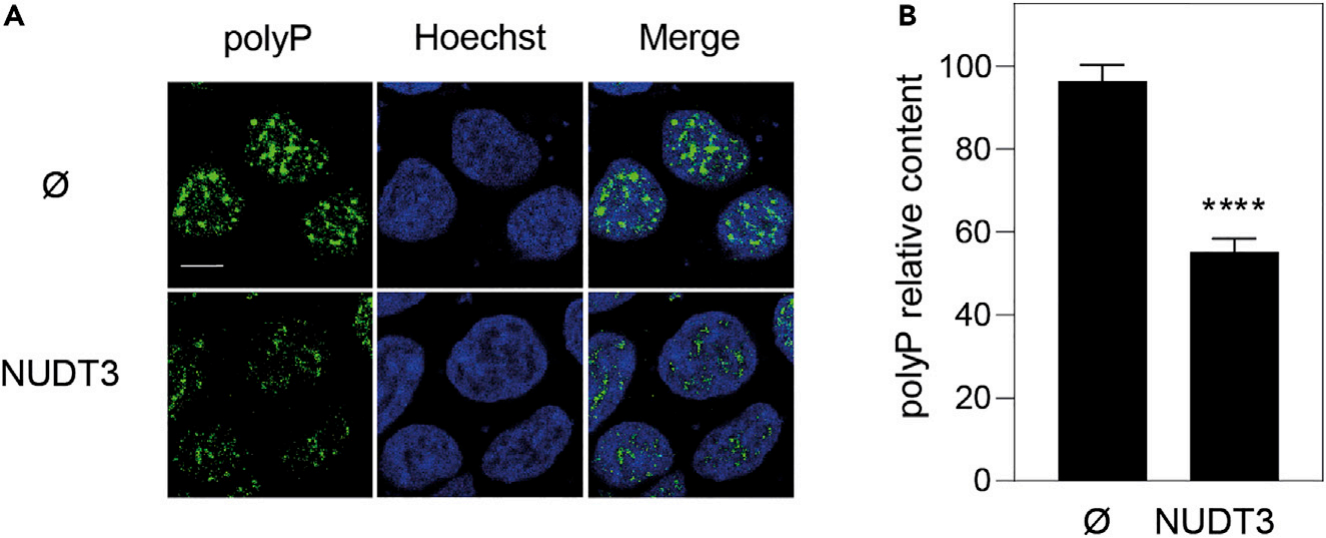
Immunolocalization of nuclear polyP using PPBD HEK293T cells transformed with pWPI empty vector (Ø) or with pWPI-NUDT3·plasmid overexpressing the polyP endopolyphosphatase (hydrolase) NUDT3 (NUDT3) for 72 h. The cells were grown and treated as described in this protocol. (A) Representative fluorescence confocal images of the above cells after being subjected to the polyP immunolocalization protocol presented in this article. Note that in this particular experiment where Nudt3 polyPase is overexpressed by transfecting cells with pWPI which contains the GFP gene as a transfection marker, the secondary antibody for the polyP immunolocalization was conjugated with Alexa 633 instead. The bar is for 10 nm. (B) Quantification of the polyP content as described here. Mean ± SEM of 5 independent experiments. A minimum of 300 cells per experiment were analyzed. Mann-Whitney test was applied. p<0.0001.

**CRITICAL:** We tried in batch purification of PPBD; for unknown reasons, we consistently obtained a staining pattern not reflecting polyP and based on a homogeneously distributed signal of tiny dots very different to the irregularly distributed pattern of several sizes dots presented in Figure 1 and Samper-Martín et al. (2021).

## Step-by-step method details

### Step 1: Preparing the cells

**Timing: 1.5 h**

This section includes the steps for culturing the cells on the coverslips and preparing them for immunostaining.

1. Coverslips preparation:
  a. Put 12 mm diameter coverslips in P24 culture plate wells.
  b. Add 90 μL of commercial poly-L-Lysine solution for 1 h (as a tip to save time, during this step, step 2 might be conducted in parallel).
  c. Wash three times using PBS.
2. Cells trypsinization:
  a. Take the culture medium carefully from the flask.
  b. Wash once using pre-warmed 5–10 mL PBS.
  c. Add 1 mL of trypsin and incubate for 3 min at the cell culture incubator.
  d. Add 4 mL of pre-warm fresh culture medium and harvest the cells. Eliminate the trypsin by centrifugation 300 × *g* for 3 min and suspend the cells in pre-warm fresh medium.

**CRITICAL:** Cells must be carefully and thoroughly suspended before counting and seeding. Clamped cells will make the ulterior fluorescence analysis very difficult.

3. Cells adhesion on the coverslip.
  a. Seed 8 × 10^4^ cell/mL in the P24 plate from step 1. Spread the cells by performing round movements assuring a homogeneous cell distribution on the coverslip.
  b. Incubate for 24 h in the cell incubator until reaching at 60%–70% confluency.

**CRITICAL:** It is essential not to go further than 70% confluence. Seeded amount will vary depending on the cell line and the experiment.

4. Cells fixation:
  a. Aspirate the culture medium carefully.
  b. Add 0.5 mL of p-formaldehyde 4% (v/v).
  c. Incubate for 15 min at 4°C.
  d. Wash three times using 0.5 mL of PBS.

**Pause point:** Coverslip containing fixed cells can be stored in PBS at 4°C for no more than a week or move forward with the immunostaining.

### Step 2: Polyphosphate immunostaining

**Timing: 20 h**

This section describes the immunostaining method for polyP detection using Xpress tagged PPBD, its visualization by confocal microscopy and further quantification.

5. Blocking and immunostaining:
  a. Blocking: Incubate the coverslip containing the cells for 1 h without shaking in 0.5 mL of blocking buffer (PBS containing goat serum 2.5% (v/v) and Tween20 0.5% (v/v)).
  b. PPBD and anti-Xpress antibody pre-incubation: mix anti Xpress antibody (5 μg/mL final concentration) and recombinant 6×His-Xpress-PPBD (20 μg/mL final concentration) in blocking buffer and incubate them for 1 h at 4°C in a rotating shaker.
  c. Remove the blocking solution and add 250 μL of the above PPDB and Xpress antibody mix. Incubate for 16 h at 4°C.
  d. Wash three times for 5 min using 0.5 mL of PBS at 20°C with gentle agitation.
  e. Incubate with 0.5 mL of the Alexa Fluor 488 or 633 secondary antibody (1:1000) in blocking solution for 1 h at 20°C in gentle agitation.
  f. Wash three times for 5 min using 0.5 mL of PBS at 20°C in gentle agitation.
  g. Hoechst staining. Add 0.5 mL of Hoechst (final concentration 1 μg/mL) in PBS for 5 min at 20°C in gentle agitation.
  h. Wash three times for 5 min using 0.5 mL of PBS at 20°C in gentle agitation.
  i. Put the coverslip upside down a slide using a drop of mounting solution. Keep them protected from light.

***Note:*** The mounting solution Fluoromount-G takes 1 h to get dry at 20°C.

### Step 3: Confocal microscopy

This section includes the microscope configuration and image acquisition. We used a Leica DMi8 microscope with TCS SP8 confocal module equipped with a Leica 63× 1.4NA HC Apo CS2 immersion oil objective. Hoechst was excited using the 405 nm laser channel. Alexa Fluor 488 or 633 were excited using its correspondent laser channel. Channel was set by using Leica Application Suite X (LASX) Acquisition software. The images were obtained by the sequential scanning named “between frames” at 400 Hz. Pixel size was 240.5 × 240.5 nm; image size was 246.03 × 246.03 μm; zoom was set at 0.75. The initial microscope settings were: pinhole 1 UA (for both excitation wavelengths), laser energy power was between 0.5 al 2%, the gain was between 700 and 900, and the offset between -10 and -20. Emission was collected using a Leica PMT detector with collection windows of 408–475 nm (405 channel), 498–550 nm (488 channel) and 648–800 nm (638 channel).These settings must be fine-tuned to obtain optimal images.

**CRITICAL:** 1. Keep the laser energy and/or the PMT gain below the maximum rendering photobleaching. 2. Take a minimum of 10 shots for every condition in three independent experiments. 3. Save the pictures in .LIF format to be open using LAS X or ImageJ Fiji. Pictures can be saved to other formats such as tiff for ulterior analysis.

### Step 4: Polyphosphate quantification

We used the free available software Fiji ImageJ 1.53c. As a summary, to quantify the polyP signal intensity we used the blue channel (Hoechst) to identify the nuclei and define the “regions of interest” (ROIs). These ROIs were the areas where polyP signal was quantified.

6. LIF file generation:
  a. Go to the ImageJ toolbar: file>open>file name>open.
  b. The window “Bio-Formats Import options” pops up; select the following options:
    i. Dataset organization>open all series.
    ii. Color options>color mode>colorized.
    iii. Split into separate windows>Split channels.
  c. Click OK to get images from the different fluorescence channels. In this case, the blue channel is for Hoechst and green for polyP.

7. ROIs selection:
  a. Select the Hoechst channel.
  b. Process>filters>Gaussian blur. Establish a value of 2 at “sigma (Radius)”. Click OK.
  c. Image>adjust>threshold. Select “dark background">default>over/under. Select the auto setting or manually move the sliders until all nuclei are delimitated.
  d. Click “apply” to obtain a binary mask.
  e. Process>binary>fill holes.
  f. Process>binary>watershed.
  g. Analyze>analyze particles. Click OK to get the ROIs.
    ***Note:*** To avoid the selection of undesired ROIs, the size of the ROI can be selected or modified to get a better automatic selection. Go to Analyze particles>size (micronˆ2). To help in a more precise selection go to Clear results>add to manager>exclude on edges.
  h. Opening “ROI manager” all the ROIs generated according to the selected parameters can be checked.
  i. Signal quantification:
  j. To eliminate unspecific signal apply a threshold (cut off around 50 in 8 bits images). Select the green (polyP) channel>image>adjust>threshold.
  k. Select “dark background”>default>over/under. Adjust the sliders manually until the unspecific signal disappear.
    **CRITICAL:** To avoid alterations in the quantification, once the threshold has been established, DO NOT click on “apply".
    **CRITICAL:** To compare the signal intensity among different quantifications (pictures, conditions, etc.).
    The threshold values must be the same.
  l. Select “ROI manager”. Click on “show all” to overlay the ROIs over the image to be quantified.
  m. Analyze>set measurements. Select at least “integrated density (intDent)” and “limit to threshold”. Click on “measure”.
  n. A new window pops up showing a table containing the results. “intDent” is the parameter used to measure the fluorescence intensity.
  o. For statistical significance, at least 400 cells in each of 3 independent experiments should be analyzed.
    We recommend the analysis of 3 independent experiments containing a minimum of 5 fields with 30–100 cells in each. For statistical significance analysis, we applied Mann-Whitney test using GraphPad Prism software.

## Expected outcomes

This protocol is for nuclear polyP staining which appears as a punctuated pattern distributed throughout the nucleus. Interestingly the intensity of the signal reduces when the endopolyPase Nudt3 is overproduced using transfected cells with the pWPI-NUDT3 plasmid (Figure 1) indicating specificity for the signal detected. For more details, see (Samper-Martín et al., 2021).

## Limitations

Cell fixation and permeabilization conditions used permit nuclear polyP visualization mainly. Polyphosphate in other places could be elusive to this method.

This protocol is for cell lines growing in flat two dimension cultures that is not how cells are in tissues or organs.

Polyphosphate quantification is by image analysis; in consequence, only polyP relative amounts can be obtained.

## Troubleshooting

### Problem 1

At the before you begin section and regarding the recombinant PPBD purification. The *E. coli* suspension remains turbid before sonication and after lysis buffer addition.

### Potential solution

Add benzonase and lysozyme again and an extra sonication cycle.

### Problem 2

At the before you begin section and regarding the recombinant PPBD purification. Äkta eluted aliquots; both in the affinity of desalting process, get a milky aspect or have some degree of precipitation.

### Potential solution

Purify PPBD again after regenerating the column by stripping all accumulated impurities (20 mM sodium phosphate, 0.5 M NaCl, 50 mM EDTA, pH 7.4) following the steps described by the provider.

### Problem 3

At the before you begin section and regarding the recombinant PPBD purification. The amount of PPBD recovered after the purification consecutively reduces.

### Potential solution

Regenerate the column by stripping all accumulated impurities (20 mM sodium phosphate, 0.5 M NaCl, 50 mM EDTA, pH 7.4) following the steps described by the provider. The column should be cleaned every 5–6 uses.

### Problem 4

At steps 2 and 3. Nuclear dotted pattern homogeneously distributed in the immunostaining.

### Potential solution

PPBD is not working properly; purify it again. In our hands, PPBD stored at −20°C lasts no more than 2 months.

### Problem 5

At steps 2 and 3. Nuclear and cytoplasmic background signal in the immunostaining including enormous randomly distributed stains.

### Potential solution

Centrifuge the secondary antibody and keep the supernatant.

## Resource availability

### Lead contact

Further information and requests for resources should be directed to the lead contact, Javier Jiménez: jjimenez@uic.es.

### Materials availability

All materials are available upon request to the lead contact, Javier Jiménez: jjimenez@uic.es.

## Data Availability

This study did not generate datasets or code.
